# Mesonephric-Like Adenocarcinoma of Uterine Corpus: A Clinicopathological and Targeted Genomic Profiling Study in a Single Institution

**DOI:** 10.3389/fonc.2022.911695

**Published:** 2022-07-05

**Authors:** Tianshi Ma, Mengyu Chai, Huafeng Shou, Guoqing Ru, Ming Zhao

**Affiliations:** ^1^Cancer Center, Department of Pathology, Zhejiang Provincial People’s Hospital, Affiliated People’s Hospital of Hangzhou Medical College, Hangzhou, China; ^2^Department of Pathology, Zhejiang Hospital, Hangzhou, China; ^3^Cancer Center, Department of Gynecology, Zhejiang Provincial People’s Hospital, Affiliated People’s Hospital of Hangzhou Medical College, Hangzhou, China

**Keywords:** mesonephric-like adenocarcinoma, uterine corpus, *KRAS*, *PIK3CA*, *PTEN*

## Abstract

**Background:**

Mesonephric-like adenocarcinoma (MLA) is a recently characterized, rare, and aggressive neoplasm that mostly arises in the uterine corpus and ovary. MLA shows characteristic pathological features similar to mesonephric adenocarcinoma of the cervix. The origin of MLA is still controversial and recognition of it remains challenging for pathologists. The aim of this study was to enrich the clinicopathological features of MLA in the uterine corpus and explore its molecular alterations by targeted next-generation sequencing (NGS).

**Methods:**

Four cases of MLA were identified among a total of 398 endometrial carcinomas diagnosed in our institution between January 2014 and December 2021. Immunohistochemistry and targeted NGS spanning 437 cancer-relevant genes were performed.

**Results:**

The most common symptom was abnormal vaginal bleeding, and the average age was 68 years. Histologically, the tumors showed a mixture of varied growth patterns including papillary, glandular, tubular, cribriform, solid, and slit-like architectures, which were lined by columnar to cuboidal cells with overlapping vesicular nuclei and sometimes nuclear grooves. Intraluminal eosinophilic colloid-like secretions were focally evident in three of the four cases. Immunohistochemically, the MLAs were positive for GATA3 (4/4), TTF-1 (3/3), luminal CD10 (3/3), calretinin (2/3), and patchy P16 (3/3) and were negative for ER (0/4) and PR (0/4). The expression of P53 was “wild type” (4/4). By targeted NGS, 3/4 (75%), 2/4 (50%), and 1/4 (25%) cases harbored *PIK3CA*, *KRAS*, and *PTEN* mutations, respectively. None of the tumors had mutations in DNA mismatch repair genes, *ARID1A/B*, *POLE*, *CTNNB1*, *SMARCA4*, or *TP53*. At the time of diagnosis, three were presented with FIGO IB stage and one with IIIC stage. Two patients received postoperative chemotherapy and radiotherapy and they were alive without evidence of disease at 8 and 56 months follow-up, respectively. One patient developed pulmonary metastasis 13 months after surgery and chemotherapy, and one was dead of the disease 24 months after the operation without adjuvant therapy.

**Conclusions:**

MLA is a rare and aggressive malignancy, representing approximately 1% of all endometrial carcinomas. It exhibits mixed architectures associated with distinctive immunophenotype and recurrent *KRAS* and *PIK3CA* mutations, supporting classified as of Müllerian origin with mesonephric differentiation.

## Introduction

Mesonephric adenocarcinoma (MA), originating from normal or hyperplastic mesonephric remnants, is an unusual and aggressive tumor most commonly occurring in the lateral walls of the cervix (1–3). Mesonephric-like adenocarcinoma (MLA) is a recently characterized, rare subtype of gynecologic carcinoma that has been included in the 5^th^ edition *World Health Organization (WHO) Classification of Female Genital Tumours* (4–6). MLA frequently arises in the uterine corpus and ovary and shares overlapping features with MA in morphology, immunophenotype, and molecular changes without the presence of mesonephric remnants (7–11). It is important to recognize MLA in the uterine corpus due to its well-established aggressive behavior (12–15). Patients frequently present at an advanced International Federation of Gynecology and Obstetrics (FIGO) stage (II to IV) with a tendency toward early recurrence and distant metastasis (12–15).

Histologically, akin to MA, MLA is characterized by a variety of growth patterns including tubular, glandular, papillary, retiform, glomeruloid, sex-cord like, and solid. Tubular and glandular patterns often predominate, and a frequent finding is the presence of small tubules containing luminal eosinophilic colloid-like materials (4, 9, 10, 13, 16). Squamous and mucinous differentiation are generally absent (9, 13, 16). The immunophenotype of MLA is also similar to MA with the expression of PAX8, GATA3, TTF1, calretinin, luminal CD10, patchy P16, and “wild type” P53, and negative staining for estrogen and progesterone receptors (4, 8, 13, 15, 16). Molecular genetic findings show both MLA and MA are characterized by recurrent *KRAS* mutations, gain of chromosome 1q, microsatellite stability, as well as alterations in chromatin remodeling genes (*ARID1A*/*B* and *SMARCA4) (* 7, 9, 10). However, accumulated evidence disclose MLAs do not infrequently harbor *PIK3CA* and *PTEN* alterations which are commonly seen in endometrial endometrioid carcinomas (EECs) but are very rare in MAs (11, 13, 16). Furthermore, extra-uterine MLAs are often associated with Müllerian lesions such as endometriosis and serous tumors (17–20). This evidence has led some pathologists to suggest that MLA may represent mesonephric-like transdifferentiation of a Müllerian tumor rather than a true mesonephric tumor (9, 11, 13, 16). Nevertheless, the definite relationship between MLA and MA has not been firmly established.

The purpose of this study was to add cases of uterine corpus MLA diagnosed at our institution with morphologic and immunohistochemical as well as detailed molecular analyses to the existing literature and to provide further evidence to support its Müllerian origination.

## Materials and Methods

### Case Selection

Among a total of 398 endometrial carcinomas (ECs) of the uterine corpus diagnosed in Zhejiang Provincial People’s Hospital between January 2014 and December 2021, four cases of MLA were identified, which accounts for approximately 1% of all ECs. The clinical details and follow-up data were obtained from a review of the electronic medical records. For all four cases, the hematoxylin-eosin (HE) stained and immunohistochemical slides were reviewed by two of the authors (TM and MZ) and the diagnosis of MLA was further confirmed according to the diagnostic criteria proposed by the recently published 5^th^ edition *WHO Classification of Female Genital Tumours* (6). This study was approved by the institutional ethics committee of Zhejiang Provincial People’s Hospital.

### Immunohistochemistry

All specimens were formalin-fixed and paraffin-embedded. Tissues were sliced into 3-μm sections. Immunohistochemistry (IHC) was performed on a Ventana Benchmark autostainer (Ventana Medical Systems, Tucson, AZ, USA). All the used primary antibodies were purchased from Beijing Zhong Shan Gold Bridge Biological Technology Co., Ltd (Beijing, China), including PAX8 (Clone EP298), GATA3 (Clone EP368), TTF1 (Clone 8G7G3/1), P53 (Clone DO-7), estrogen receptor (ER, Clone OTI1B1), progesterone receptor (PR, Clone OTI2E2), P16 (Clone 1C1), calretinin (polyclonal), and CD10 (Clone SP67). The staining process was performed in accordance with the instructions and established positive and negative controls. Stains were considered positive if there was nuclear staining for PAX8, GATA3, TTF1, P53, ER, and PR, nuclear & cytoplasmic staining for calretinin and P16, and luminal staining for CD10. Positive staining in less than 50% of tumor cells or glands was considered focally positive, and positive staining in ≥50% of tumor cells or glands was considered diffusely positive.

### Targeted Next-Generation Sequencing

Targeted NGS was performed by Nanjing Geneseeq Technology Inc. (Nanjing, China) on Illumina HiSeq4000 platform. All procedures were conducted according to the manufacturer’s protocols. This detection covers a total of about 1.53Mb nucleotide sites of exons, fusion-related introns, alternative splicing regions, and specific microsatellite sites of 437 cancer-specific genes. The sequencing results include point mutations, small indel mutations, gene fusions, copy number variations, microsatellite analysis results, and tumor mutational burden.

## Results

### Clinical Details

**Figure 1:**
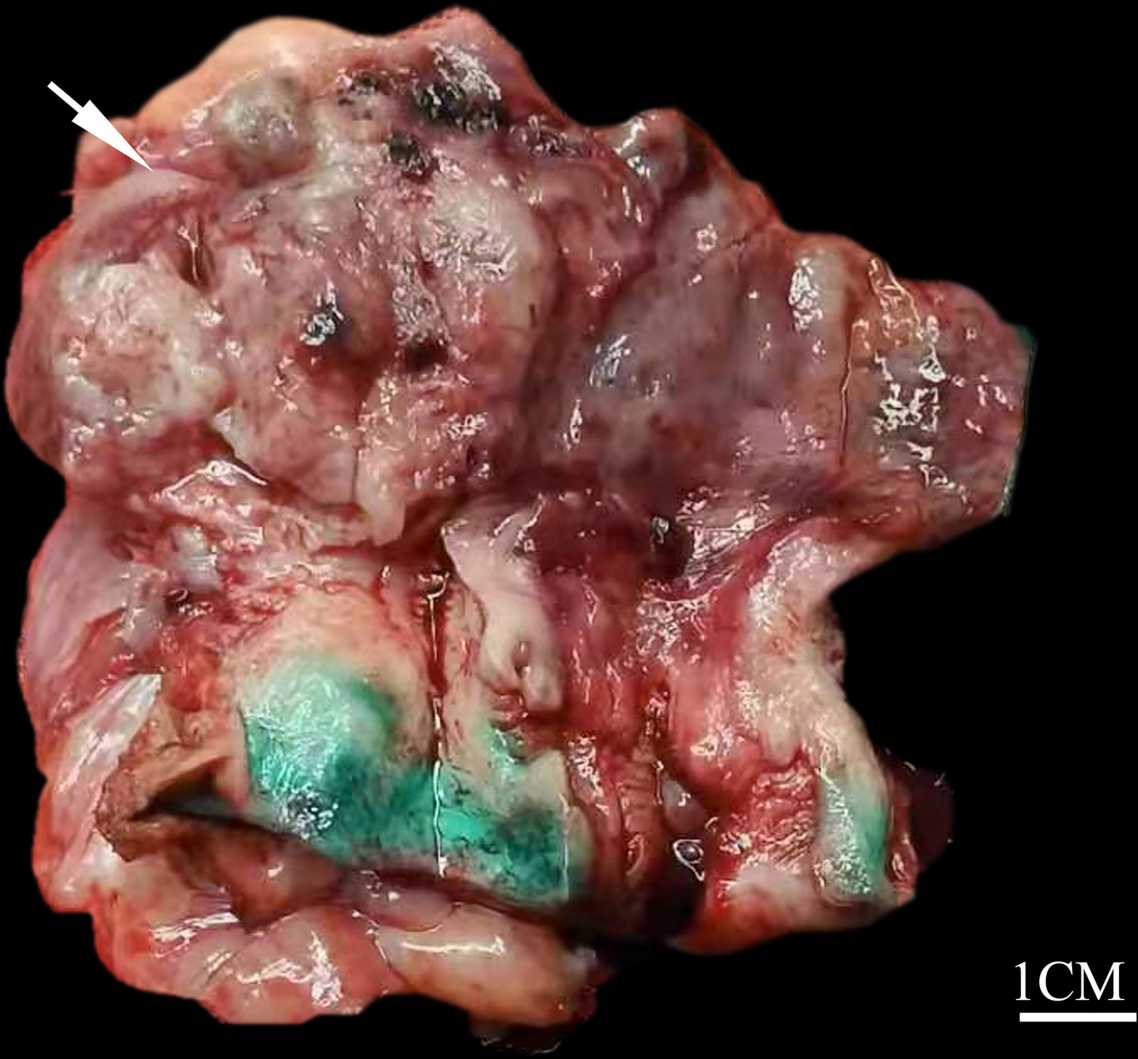
Gross findings of uterine corpus MLA. The tumor presents as a solid and soft mass with white to gray coloring (white arrow), protruding into the endometrial cavity (case 4).

The clinical details of the four patients are summarized in **Table 1**. The age at diagnosis ranged from 56 to 80 years, with an average of 68 years. Three patients were admitted to the hospital for postmenopausal bleeding with a duration from 2 months to 3 years and one presented with abdominal pain for 1 year. Radical hysterectomy and bilateral salpingo-oophorectomy were performed on all four patients and additional pelvic or para-aortic lymph node dissection was performed for three patients (cases 2-4). Grossly, all the tumors involved the uterine corpus and presented as intrauterine mass with invasion to more than one half of the myometrium. The neoplasms ranged in maximum dimension from 2 to 6 cm, with an average of 3.7 cm. Specimen sections revealed solid and soft masses that were white to gray in color **(** **Figure 1****)**. The original diagnoses included EEC (case 1), mixed carcinoma (case 2), and MLA (cases 3 and 4). At the time of presentation, three of four (75%) patients were FIGO stage IB with tumor invasion into the outer half of the myometrium and one (25%) was stage IIIC with multiple pelvic nodal metastases. After the surgery, patients 2 and 4 received both chemotherapy and radiotherapy and they were alive without evidence of disease at 56 and 8 months follow-up, respectively. Patient 3 was treated with chemotherapy and she developed pulmonary metastasis 13 months postoperatively; she subsequently underwent resection of the metastatic lesion and was disease-free after 5 months of follow-up. Patient 1 was dead of the disease (due to extensive pelvic spread) 24 months after the operation without adjuvant therapy.

**Table 1:** Clinical features of uterine corpus mesonephric-like adenocarcinomas.

Patient No.	Age	Symptoms	Size (cm)	FIGO stage	Treatments	Follow-up (mo)
1	80ys	Postmenopausal bleeding for 3 ys	6	IB	HYS+BSO	Dead of disease (24)
2	63ys	Postmenopausal bleeding for 1ys	3.3	IIIC	HYS+BSO+PL+RT+CT(carboplatin+doxorubicin)	NED (56)
3	56ys	Abdominal pain for 1ys	2	IB	HYS+BSO+PL+CT(carboplatin+taxol)	Lung metastasis (13), NED (18)
4	73ys	Postmenopausal bleeding for 2 mo	3.5	IB	HYS+BSO+PL+CT(carboplatin+doxorubicin)	NED (8)

BSO, bilateral salpingo-oophorectomy; CT, chemotherapy; HYS, hysterectomy; NED; no evidence of disease; PL, pelvic lymphadenectomy; RT, radiotherapy.

### Morphological Characteristics

**Figure 2:**
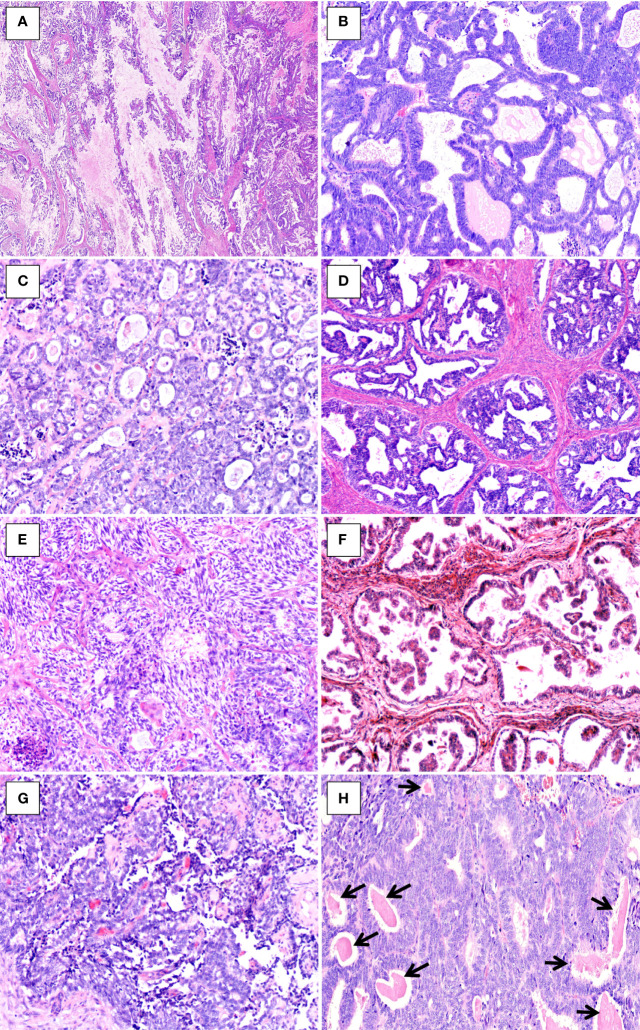
Histopathologic findings of uterine corpus MLA. **(A)** Papillary pattern with extracellular mucinous depositions (case1, HE×20). **(B)** Glandular pattern (case 4, HE×100). **(C)** Tubular pattern(case 3, HE×100). **(D)** Cribriform pattern (case 4, HE×100). **(E)** Solid pattern with spindle-shaped cells (case 3, HE×100). **(F)** Intracystic micropapillary pattern (case 2, HE×100). **(G)** Slit-like architecture (case 3, HE×100). **(H)** Dense, eosinophilic colloid-like materials (arrows, case 4, HE×100).

**Figure 3:**
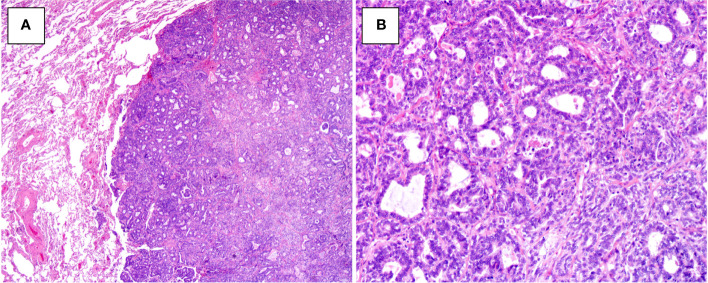
Histopathologic findings of metastatic MLA to the lung in case 3. **(A)** Well-demarcated nodular lesion (HE×20). **(B)** The lesion consists of dense tubules and glands, mimicking a primary invasive lung adenocarcinoma (HE×100).

The morphological features are illustrated in **Table 2**. Histologically, at lower magnification, the tumors showed combinations of various growth patterns, including papillary, glandular, tubular, cribriform, solid, and slit-like architectures, in a fibrous and hyalinized matrix. In the present case series, papillary (**Figure 2A**) and glandular **(** **Figure 2B****)** patterns represented the two most seen patterns, and both were presented in all four tumors. Two cases (cases 3 and 4) exhibited small tubular **(** **Figure 2C****),** back-to-back cribriform (**Figure 2D**), and solid (**Figure 2E**) patterns. Case 2 showed foci of polycystic pattern with dilated tubules and intracystic micropapillae, similar to those seen in low-grade serous carcinoma (SC) of the ovary (**Figure 2F**). Case 4 displayed focal slit-like architectures (**Figure 2G**). Dense, eosinophilic colloid-like materials (**Figure 2H**) were focally evident in the tubules in three cases. No cases had intracytoplasmic mucin or unequivocal squamous differentiation although intracystic extracellular mucinous depositions (**Figure 2A**) were noted in one case (case 1). Lung metastasis of case 3 exhibited a 0.8-cm, well-defined nodular lesion composed of tubules and glands (**Figures 3A, B****)** closely mimicking a primary invasive lung adenocarcinoma except for lacking of an *in situ* component.

**Table 2:** Histomorphological characteristics of uterine corpus mesonephric-like adenocarcinomas.

Case No.	Architectural patterns						Cytologic features			Intraluminal eosinophilic secretions	Intraluminal comedo-like necrosis	LVI
	Papillary	Glandular	Tubular	Cribriform	Solid	Slit-like	Columnar to cuboidal cells with nuclear grooves	Hobnail	Spindle			
1	80%	20%					YES	NO	NO	NO. Extracellular mucus	YES	NO
2	90%	10%					YES	YES	NO	YES	NO	YES
3	10%	10%	40%	30%	5%	5%	YES	NO	YES	YES	YES	YES
4	15%	30%	30%	20%	5%		YES	NO	YES	YES	YES	YES

LVI, lymphovascular invasion.

**Figure 4:**
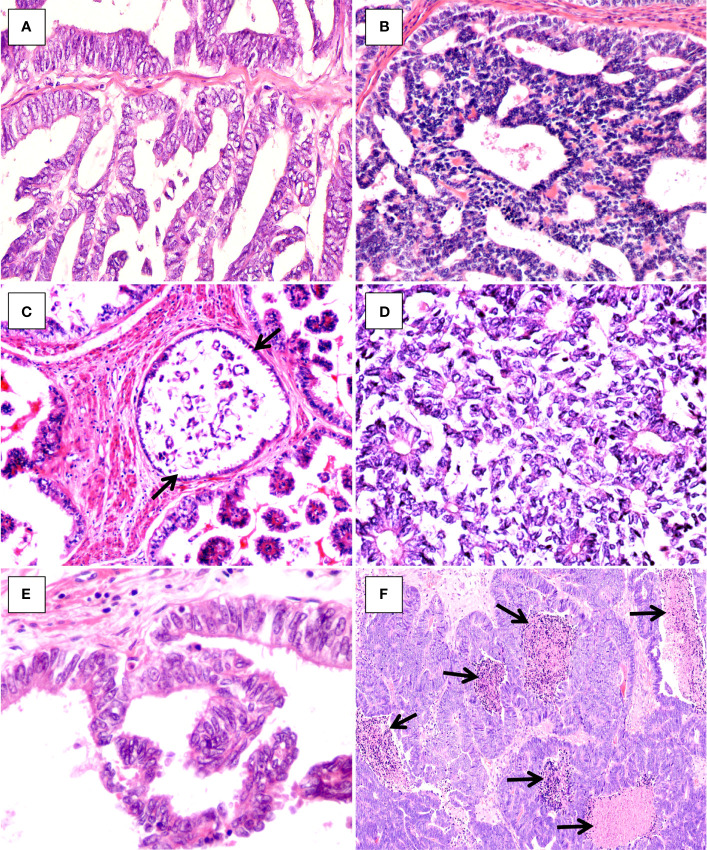
Histopathologic findings of uterine corpus MLA. **(A)** Columnar tumor cells (case1, HE×400). **(B)** Cuboidal tumor cells (case 4, HE×400). **(C)** Hobnail tumor cells (arrows, case 2, HE×300). **(D, E)** Vesicular, overlapping nuclei with nuclear grooves **(D)**, case 3, HE×300; **(E)**, case 2, HE×400). **(F)** Intraluminal comedo-like necrosis (arrows, case 4, HE×100).

Cytologically, the tumor cells were mainly columnar to cuboidal, and occasionally hobnail (**Figures 4A–C**), had scant palely eosinophilic cytoplasm, and exhibited in general moderate nuclear atypia. The nuclei were vesicular, overlapping, and sometimes angulated with nuclear grooves, resembling those seen in papillary thyroid carcinoma (**Figures 4D, E**). Spindle cells were noted in the solid areas in two cases (**Figure 2E**). Mitotic activity was conspicuous, and intraluminal comedo-like necrosis was identified in three cases (**Figure 4F**). Lymphovascular invasion was noted in three out of the four cases. For all four cases, the tumor arose in the endometrium and invaded the outer half of the myometrium with a destructively infiltrating pattern. No evidence of endometrial hyperplasia or mesonephric remnants was found, and the cervix was not involved. Twenty-three of 59 pelvic lymph nodes were positive for metastatic lesions in case 2, and no nodal metastatic lesions were identified for cases 3 or 4.

### Immunohistochemical Features

**Figure 5:**
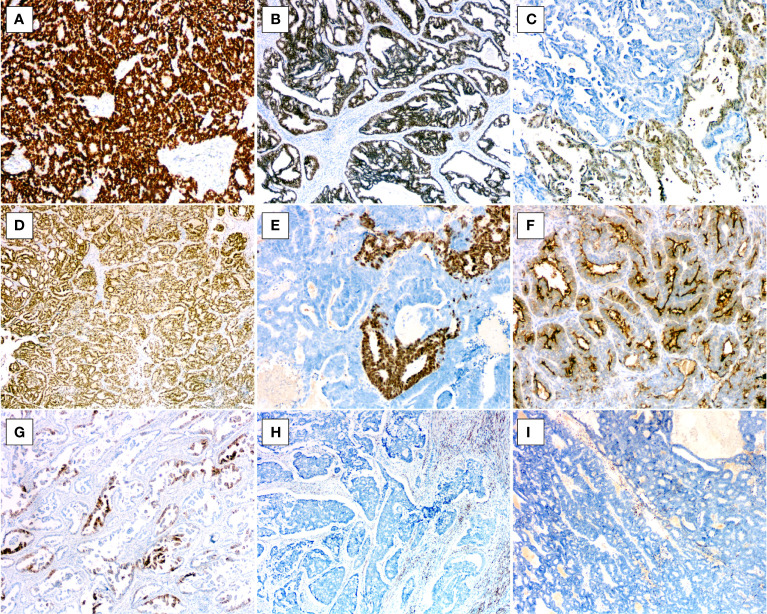
Immunohistochemical features of uterine corpus MLA. **(A)** Diffuse PAX8 positivity (case 3, ×100). **(B)** Diffuse GATA3 positivity (case 4, ×40). **(C)** Focal GATA3 positivity (case 1, ×100). **(D)** Diffuse TTF1 positivity (case 3, ×40). **(E)** Focal TTF1 positivity (case 4, ×100). **(F)** Luminal CD10 positivity (case 3, ×100). **(G)** Focal calretinin positivity (case 2, ×40). **(H)** ER negativity (case 3, ×40). **(I)** PR negativity (case 4, ×40).

**Figure 6:**
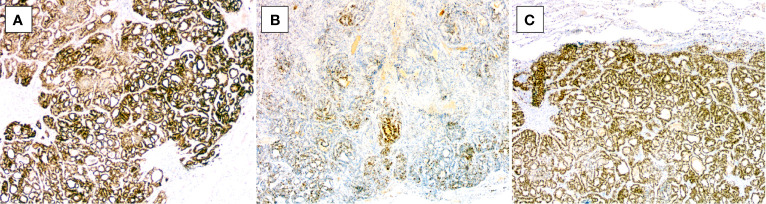
Immunohistochemical findings of metastatic MLA to the lung in case 3. The tumor cells are positive for **(A)** PAX8 (×40), **(B)** GATA3 (×40), and **(C)** TTF1 (×40).

The immunohistochemical results are shown in **Table 3**. Not every case was stained for all markers. In all cases tested, the tumor cells were positive for PAX8 (2/2, diffusely in both) (**Figure 5A**), GATA3 (4/4, diffusely in two and focally in two) (**Figures 5B, C**), and TTF1 (3/3, focally in two and diffusely in one) (**Figures 5D, E**), and the one (case 3) which was diffusely positive for TTF1 also diffusely expressed GATA3. Three of three tumors showed focal luminal expression of CD10 (**Figure 5F**), and two out of three exhibited focal calretinin positivity (**Figure 5G**). Patchy P16 expression was noted in three cases analyzed. All cases exhibited a wild-type pattern of P53 expression, and all tumors were negative for both ER (**Figure 5H**) and PR (**Figure 5I**). For the metastatic MLA in the lung in case 3, the tumor cells were positive for PAX8, GATA3, and TTF1 (**Figures 6A–C****)**, in accordance with the immunoprofile of the primary tumor of the uterine corpus.

**Table 3:** Immunohistochemical characteristics of uterine corpus mesonephric-like adenocarcinomas.

Case No.	ER/PR	PAX8	GATA3	TTF-1	CD10 (luminal)	P53	P16	Calretinin
1	-/-	ND	focally +	ND	ND	WT	ND	ND
2	-/-	ND	focally +	focally +	focally +	WT	patchy+	focally +
3	-/-	diffusely +	diffusely +	diffusely+	focally +	WT	patchy+	focally +
4	-/-	diffusely +	diffusely +	focally +	focally +	WT	patchy+	focally +

WT, wild type; ND, not done; +, positive; -, negative.

### Molecular Genetic Findings

**Figure 7:**
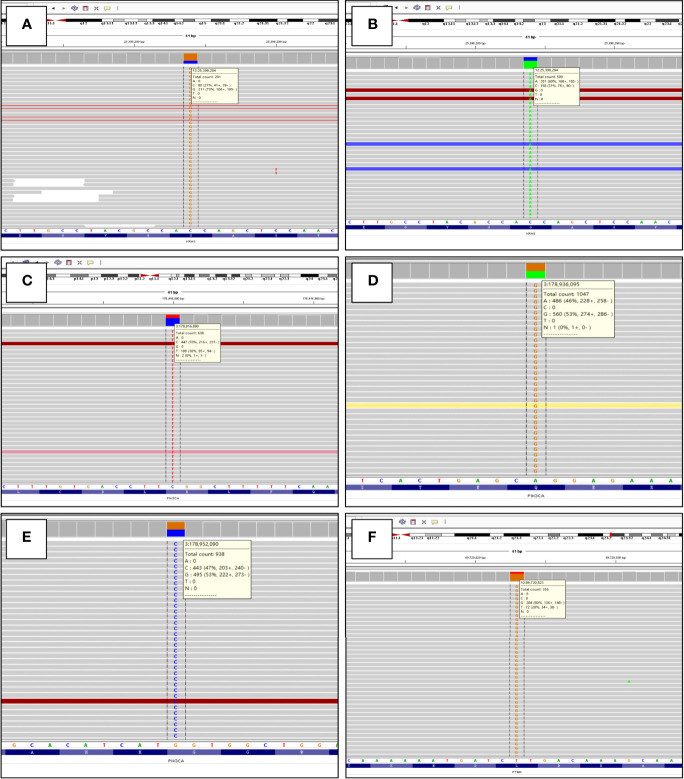
Molecular genetic findings of uterine corpus MLA detected by targeted next-generation sequencing, as shown by the Integrative Genomics Viewer screenshot. **(A)** *KRAS* exon2: c.35G>C (p.G12A) (case 1). **(B)** *KRAS* exon2: c.35G>T (p.G12V) (case 2). **(C)** *PIK3CA* exon1: c.277C>T (p.R93W) (case 2). **(D)** *PIK3CA* exon9: c.1637A>G (p.Q546R) (case 3). **(E)** *PIK3CA* exon20: c.3145G>C (p.G1049R) (case 4). **(F)** *PTEN* exon8: c.974T>G (p.L325R) (case 1).

The molecular genetic findings are listed in **Table 4**. Genetic testing using targeted NGS spanning 437 cancer-relevant genes was performed on all four cases. The tumor mutational burdens were overall low and varied from 2.1 to 7.4 mutations per megabase. A total of 14 genes with 18 mutations were identified across all cases and contained 14 missense, one nonsense, one frameshift, one nonframeshift deletion mutation, and one splice site mutation. Five genes (*KRAS*, *PIK3CA*, *PTEN*, *ATM*, *MYCN*) of the mutations have been previously reported in MLA. *KRAS* activating mutations were found in two of four (50%) cases harboring G12A (case 1) and G12V (case 2) (**Figures 7A, B**), and *PIK3CA* mutations (all missense mutations) were identified in three of four cases (75%) harboring alterations including R93W (case 2), Q546R (case 3), and G1049R (case 4) (**Figures 7C–E**). Case 2 had concurrent mutations of *KRAS* and *PIK3CA. PTEN* missense mutation (L325R) (**Figure 7F**) was identified in case 1 which was accompanied by *KRAS* mutation. Both the two *KRAS-*nonmutated tumors harbored *PIK3CA* mutations (cases 3 and 4). Copy number variation was only identified in case 3, which exhibited a gain of *NKX2-1* (TTF1, copy number, CN: 4.2). None of the tumors had mutations in DNA mismatch repair genes, *ARID1A/B*, *POLE*, *CTNNB1*, *SMARCA4*, or *TP53*.

**Table 4:** Molecular genetic findings of uterine corpus mesonephric-like adenocarcinomas.

Case No.	Case 1	Case 2	Case 3	Case 4
Mutations	PTEN: exon 8 c.974T>G(p.L325R). VAF:78.85% Uncertain significance	DUSP2*:exon2* c.504C>G(p.Y168*). VAF:77.55% Uncertain significance	PIK3CA:exon9 c.1637A>G(p.Q546R). VAF:52.6% Likely pathogenic	PIK3CA:exon20 c.3145G>C(p.G1049R). VAF:45.9% Likely pathogenic
	KRAS: exon2 c.35G>C(p.G12A). VAF:72.29% Likely pathogenic	KRAS: exon2 c.35G>T(p.G12V). VAF:69.45% Likely pathogenic	SMARCB1:exon8 c.1091_1093del(p.K364del).VAF:14.87% Uncertain significance	CHD4:exon25 c.3740T>C(p.I1247T). VAF:2.03% Uncertain significance
	ATM:exon50 c.7466C>T(p.S2489F). VAF:35.14% Uncertain significance	SMAD4:exon8 c.913C>A(p.H305N).VAF:31.61% Uncertain significance	MYCN:exon2 c.131C>T(p.P44L). VAF:11.52% Uncertain significance	
	ATM:exon10 c.1372_1382dup (p.E461Dfs*16). VAF:21.69% Uncertain significance	PIK3CA:exon1 c.277C>T(p.R93W). VAF:29.91% Uncertain significance		
	KMT2C:intron7 c.1012+1G>A. VAF:8.67% Uncertain significance	DPYD:exon21 c.2737A>G(p.I913V). VAF:23.62% Likely benign		
	TMPRSS2:exon3 c.230A>C(p.H77P). VAF:8.11% Uncertain significance	PTCH1:exon23 c.4282G>A(p.E1428K).VAF:22.68% Uncertain significance		
		TACC3:exon9 c.1787G>A(p.R596Q). VAF:20.33% Uncertain significance		
CNV			**NKX2-1 (**CN:4.2)	
TMB (per megabase)	7.4 mutations	7.4 mutations	3.2 mutations	2.1 mutations
Microsatellite analysis	No mutations in DNA mismatch repair genes were detected			

CN, copy number; CNV, copy number variation; TMB, tumor mutational burden; VAF, variant allele frequency.

## Discussion

In this study, we used previously characterized knowledge of the morphologic and immunohistochemical features of MLAs to retrospectively identify these tumors in the archives of our institution. Among a total of 398 ECs diagnosed in our institution between January 2014 and December 2021, four cases of MLA were retrieved, accounting for approximately 1% of all ECs, in accordance with the recently reported prevalence of 0.7% (4/570) by Kolin et al. in 2019 (16), and 0.7% (2/300) by Mills et al. in 2022 (21). Despite being very rare, uterine corpus MLA may be under-recognized due to its diverse morphology and close resemblance to other commonly encountered endometrial cancers such as EEC, clear cell carcinoma (CCC), SC, and carcinosarcoma. In fact, for the four cases presented here, only two had been correctly diagnosed as MLA originally, with the other two diagnosed as EEC and mixed carcinoma, respectively. Similarly, in the retrospective case series study by Euscher et al. (13), of the 23 cases of uterine corpus MLA analyzed (most were referral cases), 18 were initially classified as carcinomas other than MLA, including EEC in 13 cases, adenocarcinoma not otherwise specified in four cases, and SC in one case.

The four cases of uterine corpus MLA presented here showed morphologic, immunohistochemical, and molecular features consistent with those in previously described cases (**Table 5**). In general, the most useful morphological clues for diagnosing MLA include: a variety of growth patterns seen in combination and within the same tumor; the presence of distinctive nuclear features that resemble those seen in papillary thyroid carcinoma, such as nuclear grooves, nuclear overlap, and open chromatin; the presence, at least focally, of densely eosinophilic intraluminal secretions in the tubules; and the lack of marked nuclear atypia and pleomorphism, and unequivocal squamous differentiation (4, 9, 10, 13, 16). Foci of solid architecture composed of spindle cells can be noted in MLAs, which should not be regarded as a sign of sarcomatoid differentiation, given the lack of heterologous differentiation in these areas and the consistent absence of *TP53* mutations in these tumors (16). MLAs typically have no intracytoplasmic mucin frequently seen in EECs but they can contain mucin-like material within the luminal spaces 13). Rare features that have been documented in MLAs include sex cord-like/trabecular pattern reminiscent of the corded and hyalinized variant of EEC (22), papillary pattern covered by hobnail-type cells or cytoplasmic clearing cells mimicking CCC (13), and dedifferentiation to sheets of anaplastic discohesive cells (23). In addition, EC with architectural and cytologic features suggesting low-grade SC of the ovary (such as polycystic pattern with dilated tubules and intracystic micropapillae, as two of our cases have illustrated), should also prompt consideration of MLA. In three out of the four MLAs, intraluminal comedo-like necrosis resembling that seen in intraductal carcinoma of the breast was noted; this morphology has previously been documented in a subset of MLAs (13). While careful attention to histology can help flag tumors for potential mesonephric-like classification, ultimately ancillary studies are often required for confident classification as MLA. In this study, for all cases tested, the tumor cells are positive for PAX8, GATA3, TTF1, and luminal CD10, and are negative for ER and PR, consistent with the immunohistochemical results previously reported. Pors et al. (8) compared the sensitivity and specificity of GATA3, TTF1, CD10, and calretinin in the diagnosis of MLAs and reported GATA3 to be the best overall marker, but staining can be weak to moderate in intensity and positive in only a minority of cells (<10%). In their study, TTF1 had high sensitivity and specificity comparable to GATA3 for MLAs, however, it was expressed in only 12.5% of MAs, suggesting MLA and MA may be biologically different entities. TTF1 and GATA3 can show an inverse staining pattern in a minority of MLAs (8). It is worth noting that both GATA3 and TTF1 can be seen in 6% and 1% of non-MLA ECs, respectively (8). Previous studies have found that both TTF1 and GATA3 expression are poor prognostic factors for ECs, and some of these TTF1 or GATA3-positive cases may in fact represent MLAs (24, 25). Euscher and colleagues (13) investigated the immunophenotypic features in 23 cases of MLA of the endometrium and found similar results. In their study, 15/16 (94%) and 11/16 (69%) of cases expressed GATA3 and TTF1, respectively, and the intensity and number of cell staining of GATA3 were greater than that of TTF-1 in most cases. These authors suggested that a panel of immunohistochemical stains should be employed to facilitate a diagnosis of MLA and included first-line markers GATA3, TTF1, ER, and PR, with CD10 and calretinin serving as supplemental immunohistochemical stains. Most recently, Kim et al. (15) found that in 25 cases of endometrial MLA, 68% showed at least moderate nuclear GATA3 immunoreactivity in more than 25% of tumor cells, and 81% of examined cases expressed TTF1 often in a heterogeneous pattern. In that study, all cases expressed at least one of the two markers, with an inverse staining pattern of GATA3 and TTF1 noted in 30% of cases (15). Loss of ER is typically observed in MLAs, however, this is not a universal finding among reported cases. In the study by Euscher et al. (13), focal positivity of ER (usually in less than 10% of tumor cells) can be seen in up to 30% of MLAs. Hence some positivity of ER does not necessarily preclude the diagnosis of MLA. PR was negative in almost all reported cases; thus, it can be concluded that PR is a more reliable negative marker for MLA (5).

**Table 5:** The most useful histopathologic and molecular genetic features for the diagnosis of uterine corpus mesonephric-like adenocarcinomas.

Morphological features	(1) In combination of a variety of growth patterns including tubular, glandular, papillary, retiform, glomeruloid, sex-cord like, spindle cells, and solid; (2) Columnar to cuboidal cells with distinctive nuclear features resembling those seen in papillary thyroid carcinoma, such as nuclear grooves, nuclear overlap, and open chromatin; (3) The presence, at least focally, of densely eosinophilic intraluminal secretions in the tubules; (4) The lack of marked nuclear atypia and pleomorphism, and unequivocal squamous differentiation.
Immunohistochemical features	(1) Mostly positive for GATA3 and TTF1, and less commonly for calretinin and luminal CD10; (2) Patchy P16 and “wild type” P53 expression; (3) Negative for estrogen receptor (can be focally positive) and progesterone receptor.
Molecular genetic features	Somatic *KRAS* mutations (in up to 90% cases), with or without mutations in *PIK3CA*, *PTEN*, and *CTNNB1.*

Increasing use of mutational analysis techniques in an effort to improve understanding of tumor biology and identify potential therapeutic targets has led to the identification of recurrent *KRAS* mutations in MLAs. Although results from smaller series suggest that *KRAS* mutation is a consistent finding among MLAs, several previously reported larger series reported *KRAS* mutation in 76 to 89% of cases (10, 11, 13). All the cases lacking *KRAS* mutation had histologic and immunohistochemical features diagnostic of MLA. In this study, *KRAS* activating mutations were only found in two of four (50%) cases harboring G12A (case 1) and G12V (case 2), respectively. In our study, the lower proportion of *KRAS* mutation may be related to the small number of samples included. Our two cases without a *KRAS* mutation both had histologic features of MLA as well as diagnostic immunohistochemical support including expression of GATA3 and TTF1. In most of the reported cases in the literature, MLAs have only had mutations in *KRAS*, typically affecting the hotspot codon 12 including G12D, G12V, G12C, and G12A (9–13, 15). A small number of cases have been noted in the literature with concurrent genetic alterations, including mutations in *PIK3CA*, *PTEN*, and *CTNNB1* (9, 10, 13, 16), which are characteristic molecular alternations of EECs (26). Among the small number of endometrial MLAs without a *KRAS* mutation reported, one harbored a mutation involving *BRAF*, another gene in the RAS/MAPK pathway (11), and one had mutations in *PIK3CA*, *PTEN*, and *CTNNB1 (* 13). Our findings reflect the literature experience. Of the two cases with a *KRAS* mutation, both had additional mutations, including *PIK3CA* and *PTEN* in each case. For the two cases lacking *KRAS* mutation, both had mutations in *PIK3CA*, and one of which had a gain of *NKX2-1* (TTF1, copy number, CN: 4.2), which had previously been reported in MLAs (9). The presence of concomitant *PIK3CA* and/or *PTEN* mutations in some cases of endometrial MLA has led to the hypothesis that these tumors are Müllerian or endometrioid derived but differentiating along mesonephric lines, representing a distinctive variant of EC (9, 11, 13, 16). The findings of our study lend further support to this assertion.

Although *KRAS* mutations had been identified in the vast majority of reported MLAs, a *KRAS* mutation is usually not required for the diagnosis of MLA. Furthermore, *KRAS* mutation is not unique to MLA in endometrial cancer. In their evaluation of 570 ECs which had undergone molecular profiling, Kolin et al. (16) have identified *KRAS* mutation in up to17% (98/570) of ECs, in which most were endometrioid histotype, followed by carcinosarcoma (nine cases), and SC and MLA (both were four cases). Thus, the diagnosis of the MLA variant of EC remains based primarily on histologic and immunohistochemical grounds. The main differential diagnosis of MLA is EEC, and many of the previously reported endometrial MLA were initially mistaken for EEC (13, 14). This is not surprising given that the various architectural patterns characteristic of MLAs may also occur in EECs. Features in favor of MLA include a lack of endometrioid intraepithelial neoplasia in the background, absence of unequivocal squamous differentiation and intracytoplasmic mucin, and identification of cytological features of nuclei with vesicular chromatin and nuclear grooves. Positive immunoreactivity for GATA3 and TTF1 and negativity for ER and PR support the diagnosis of MLA. When a micropapillary architectural pattern in association with high-grade nuclear atypia is observed, SC should be considered in the differential diagnosis. SC is characterized by mutation-type p53 immunostaining pattern and p16 block-staining, features that are never seen in MLA. ER and PR are not helpful in differentiating because both MLA and SC share negative hormone receptor expression. The occurrence of admixtures of architectural patterns and, rarely, a papillary pattern covered by hobnail-type cells or cytoplasmic clearing cells sometimes renders it difficult to differentiate MLA from endometrial CCC. Although expression of HNF1β in carcinomas of mesonephric origin suggests this marker could be of limited use in distinguishing MLA from CCC, the combination of napsin A and Alpha methyacyl CoA racemase can be helpful for their distinction, as both markers are usually diffusely expressed in CCCs and only focally expressed in a minority of MLAs (27). Lastly, as mentioned above, the lack of heterologous differentiation and the consistent absence of *TP53* mutations can distinguish MLAs containing solid areas with spindle cells from Müllerian carcinosarcomas. For metastatic MLA to the lung, the positive TTF1 staining and negative hormone receptors staining can be confused with a primary lung adenocarcinoma. In patients with a history of gynecological malignancy, one should always keep in mind to perform PAX8 staining. The presence of small tubules with eosinophilic secretions and lack of an *in situ* component provide morphological clues to metastatic MLA. Additional staining for GATA3, luminal CD10, and calretinin can further help support the diagnosis. Furthermore, metastatic MLA in the lung may closely resemble a metastatic thyroid carcinoma (28), given their shared histologic and immunophenotypic features (e.g., nuclear grooves, eosinophilic secretions within tubules, positive staining of PAX8 and TTF1, and negative staining of hormone receptors). In this setting, adding IHC for GATA3 and thyroglobulin may help distinguish the two lesions, however, comprehensive clinicopathologic correlation is critical for the distinction.

Although MLAs are difficult to diagnose, their accurate identification is important due to their relatively poor prognosis. According to the previous reports, MLA displays an aggressive biological behavior with more than half of the published cases presenting with advanced stage (FIGO≥II stage) at diagnosis (12). It is associated with a considerable risk of recurrent disease with a tendency to metastasize beyond the pelvis; especially to the lungs. Euscher et al. (13) have disclosed several frequent adverse prognostic indicators in uterine corpus MLAs, including large tumor size, deep myometrial invasion, cervical stromal invasion, and lymphovascular invasion, as compared with other histologic variants of EC, such as EEC and SC. In that study, the authors also documented that MLA was an independent risk factor for adverse patient outcomes and showed shorter progression-free survival and overall survival compared to low-grade EEC as well as SC (13). The aggressive biology of MLA has further been confirmed by two recently published large series of studies. In the first study that included 44 MLAs of the uterine corpus and 25 MLAs of the ovary, 59% of the former and 42% of the latter developed recurrences, most commonly distant. The 5-year disease-specific survival (DFS) for MLA of the uterine corpus and ovary was 72% and 71%, respectively (14). In the second study including 25 MLAs of the uterine corpus, among the 23 cases with follow-up periods of more than 3 months, 72% showed local or metastatic recurrences, with a mean DFS of 22.6 months (15). In our study, three of the four patients presented with advanced-stage disease, developed tumor metastasis, or died of the disease. On the basis of integrated genomic characterization, endometrial cancers are classified into four molecular groups indicating stratified prognosis (26). Since MLAs have an absence of *POLE* mutation, *TP53* mutation, and mismatch repair-deficient, they belong to the group with no specific molecular profile and are probably responsible for the proportion of poor prognosis in this group.

Given their marked propensity for aggressive behaviors, and given that patients who receive surgery for early-stage disease have a tendency to suffer a recurrence and metastasis, previous studies have recommended that uterine MLAs should not be graded by the FIGO grading system but should all be automatically regarded as high-grade and therefore treated by algorithms utilized for high-grade ECs (13, 14). Because of its rarity, there are not yet clear clinical guidelines for the management of MLA, and the efficacity of adjuvant radiation and/or chemotherapy remains largely unknown (12). However, a recently published report showed sustained response to a combination of lenvatinib and pembrolizumab in two patients with recurrent *KRAS*-mutated MLAs (29), providing evidence that uterine corpus MLAs may merit a specialized therapeutic approach. There may be a role for targeted inhibitors of the RAS/MAPK pathway in MLAs, given their underlying *KRAS* mutations and a clinical study had shown that thyroid carcinomas with *KRAS* mutations were very sensitive to lenvatinib (30). Furthermore, because the current standard of surveillance strategies for EC patients usually does not include chest imaging, some authors have proposed chest imaging could be prospectively incorporated into the surveillance strategies for patients with MLAs, given the reported high risk for pulmonary metastases, whether at diagnosis or during disease progression (21).

## Conclusions

In summary, our study confirms that MLA is a rare and aggressive malignancy, representing approximately 1% of all ECs. It exhibits mixed architectures associated with distinctive immunophenotype and recurrent *KRAS* and *PIK3CA* mutations. Our findings provide further evidence to support the concept that uterine corpus MLA is derived from a Müllerian substrate with differentiation along mesonephric lines. Increasing prospective and retrospective recognition of this unusual variant of EC is critical to better understanding the clinical and therapeutic implications of this diagnosis.

## Data Availability Statement

The original contributions presented in the study are included in the article/supplementary material, further inquiries can be directed to the corresponding author/s.

## Ethics Statement

The studies involving human participants were reviewed and approved by the Institutional Review Board Committee of Zhejiang Provincial People’s Hospital, Affiliated People’s Hospital of Hangzhou Medical College. Written informed consent for participation was not required for this study in accordance with the institutional requirements.

## Author Contributions

TM, MC, and MZ conceptualized the study concept and design. HS provided the follow-up information and analyzed the clinical data. TM and MZ were in charge of the histologic diagnosis and interpretation of immunohistochemistry and analysis of the results of the targeted next-generation sequencing. TM and MC drafted the manuscript, and MZ and GR revised the manuscript. All authors contributed to the article and approved the submitted version..

## Funding

This work was supported by Zhejiang Provincial Natural Science Foundation (LY21H160052), and Zhejiang Provincial Medicine and Health Research Foundation (2019KY020, 2019KY029, 2021KY535). The funders did not have any role in the design and conduct of the study, the analysis and interpretation of the data, or the preparation of the manuscript.

## Conflict of Interest

The authors declare that the research was conducted in the absence of any commercial or financial relationships that could be construed as a potential conflict of interest.

## Publisher’s Note

All claims expressed in this article are solely those of the authors and do not necessarily represent those of their affiliated organizations, or those of the publisher, the editors and the reviewers. Any product that may be evaluated in this article, or claim that may be made by its manufacturer, is not guaranteed or endorsed by the publisher.
